# Supplementary material for: Temporal lineage replacements and dominance of imported variants of concern during the COVID-19 pandemic in Kenya
Source: Commun Med (Lond). 2022 Aug 17;2:103. doi: 10.1038/s43856-022-00167-8 (PMC9382597; doi:10.1038/s43856-022-00167-8)
Supplement: Supplementary file 1 — Supplemental Information [file 43856_2022_167_MOESM1_ESM.pdf]

Supplementary Table 1. SARS-CoV-2 Pango lineages detected during the 5 COVID-19 waves in Kenya.

Pango Lineage	Wave 1	Wave 2	Wave 3	Wave 4	Wave 5	Description
B.1	72	45	4			*European Lineage*
B.1.1	27		2			*European Lineage*
A.25	8					*Ugandan Lineage*
B.1.243	3					*USA Lineage*
B.1.393	3					*UG/KY*
B.1.549	2	11				*Kenyan Lineage*
B.1.1.1	2					*England*
B.1.1.356	2					*USA Lineage*
A	1					*Root*
AY.16			65	217	1	*Delta VoC sublineage - Kenya and other countries*
AY.46			1	61	7	*Delta VoC sublineage - Africa lineage*
B.1.1.7			166	26		*Alpha VoC*
B.1.617.2				12		*Delta VoC - Predominantly Indian lineage*
AY.116				7	1	*Delta VoC sublineage - Africa lineage*
AY.122				6		*Delta VoC sublineage - European lineage*
AY.46.4				6		*Delta VoC sublineage - USA lineage*
AY.61				5		*Delta VoC sublineage - Italy lineage*
AY.65				5		*Delta VoC sublineage - Baharain lineage*
AY.71				2		*Delta VoC sublineage - European lineage*
B.1.351			12	1		*Beta VoC*
AY.120.2			1	1		*Delta VoC sublineage - South Africa lineage*
AY.109				1		*Delta VoC sublineage - Nigerian lineage*
AY.126				1		*Delta VoC sublineage - European lineage*
AY.16.1				1		*Delta VoC sublineage - France lineage*
AY.41				1		*Delta VoC sublineage - European lineage*
AY.9.2				1		*Delta VoC sublineage - European lineage*
B.1.525			19			*Eta VoC*
A.23.1		3	10			*Former VoI - East African Lineage*
B.1.530		8	2			*Kenyan Lineage*
AY.10			1			*Delta VoC sublineage - UK lineage*
B.1.12			1			*Luxembourg lineage*
B.1.596.1		3				*Kenyan Lineage*
A.23		2				*Ugandan Lineage*
B.1.1.254		2				*South African Lineage*
B.1.428		2				*Danish Lineage*
B.1.340		1				*USA Lineage*
B.1.596		1				*USA Lineage*
N.8		1				*Kenyan Lineage*
BA.1.1					162	*Omicon VoC*
BA.1					26	*Omicon VoC*
Lineage Count	120	79	284	354	197	

VoC = Variant of Concern, Description source = https://cov-lineages.org/lineage_list.html

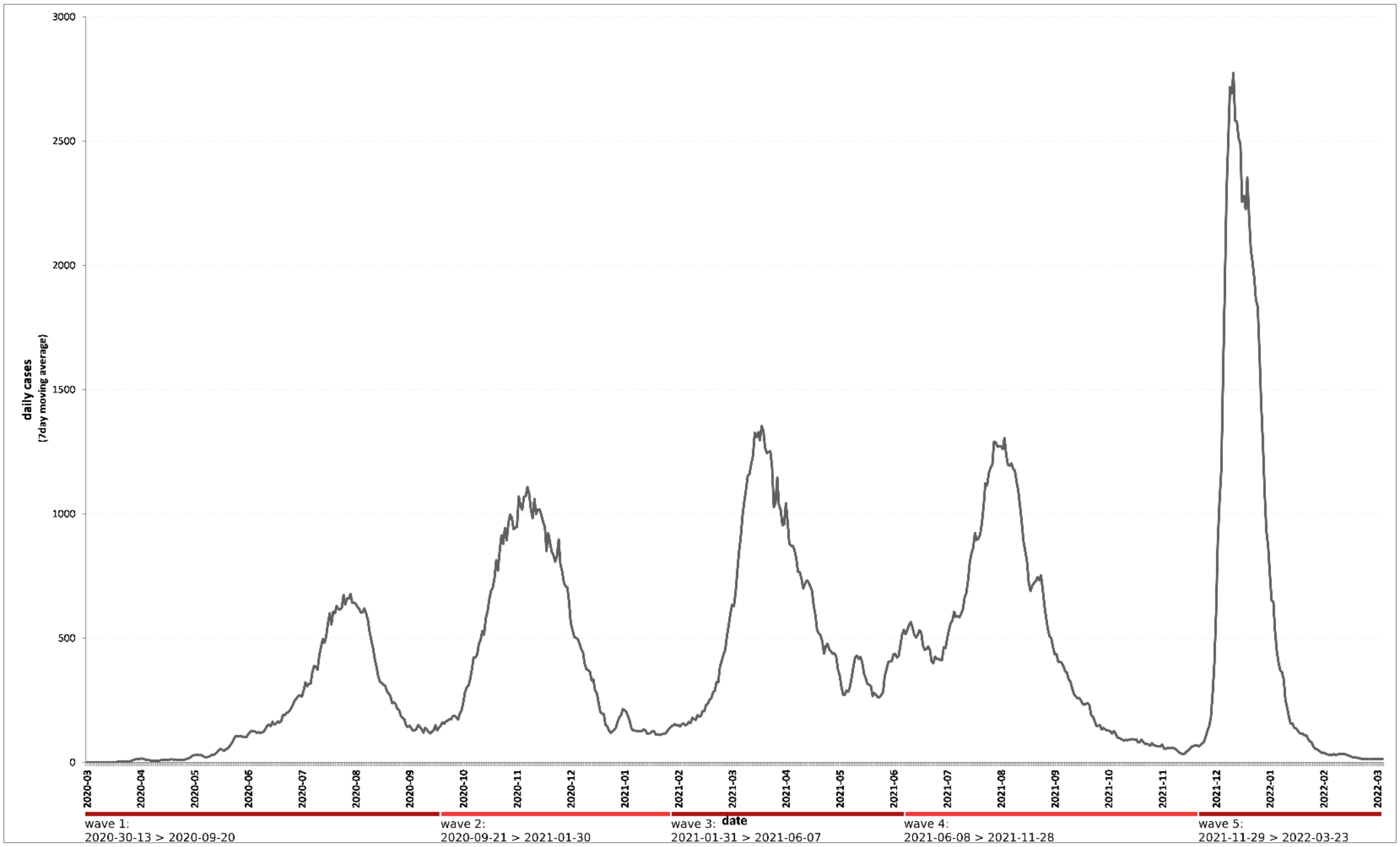

Supplementary Figure 1. 7 day moving average plot showing daily COVID-19 cases in Kenya between March 2020 and March 2022. Bars below dates shaded in red stratify the five COVID-19. The raw data for this plot was sourced from https://covid.ourworldindata.org/data/owid-covid-data.csv.

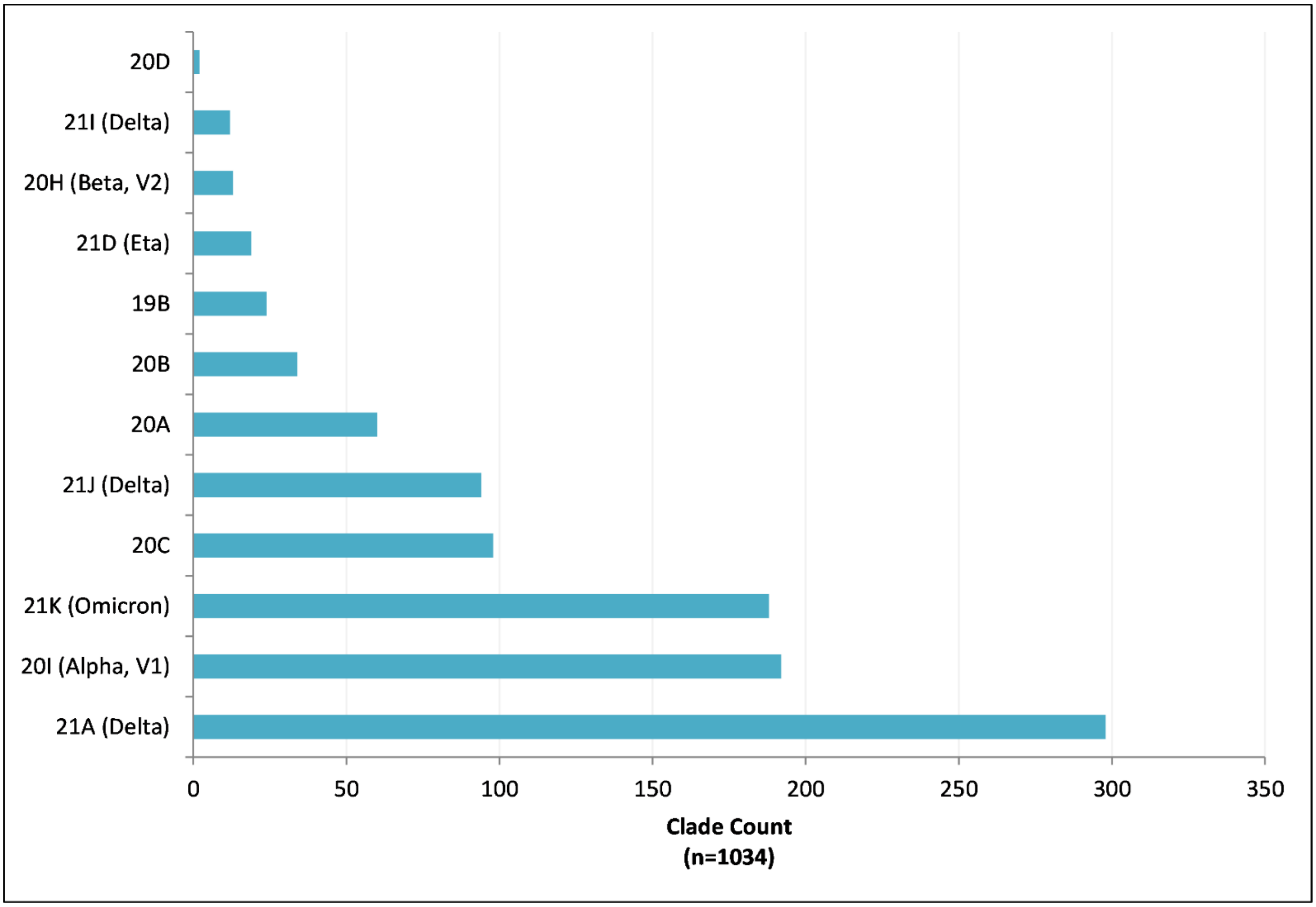

Supplementary Figure 2. Nextstrain Clade frequencies from our dataset (n=1034). The Delta clade 21A was the most frequent clade, followed by 20I Alpha and 21K Omicron clades. Clade 20D was the least frequent observed clade.
