# Supplementary material for: Temporal lineage replacements and dominance of imported variants of concern during the COVID-19 pandemic in Kenya
Source: Commun Med (Lond). 2022 Aug 17;2:103. doi: 10.1038/s43856-022-00167-8 (PMC9382597; doi:10.1038/s43856-022-00167-8)
Supplement: Supplementary file 3 — Reporting Summary [file 43856_2022_167_MOESM3_ESM.pdf]

nature portfolio

Corresponding author(s): John Waitumbi

Last updated by author(s): Jul 12, 2022

Reporting Summary

Nature Portfolio wishes to improve the reproducibility of the work that we publish. This form provides structure for consistency and transparency in reporting. For further information on Nature Portfolio policies, see our Editorial Policies and the Editorial Policy Checklist.

Statistics

For all statistical analyses, confirm that the following items are present in the figure legend, table legend, main text, or Methods section.

n/a	Confirmed	
☒	☐	The exact sample size (*n*) for each experimental group/condition, given as a discrete number and unit of measurement
☒	☐	A statement on whether measurements were taken from distinct samples or whether the same sample was measured repeatedly
☒	☐	The statistical test(s) used AND whether they are one- or two-sided<br>*Only common tests should be described solely by name; describe more complex techniques in the Methods section.*
☒	☐	A description of all covariates tested
☒	☐	A description of any assumptions or corrections, such as tests of normality and adjustment for multiple comparisons
☒	☐	A full description of the statistical parameters including central tendency (e.g. means) or other basic estimates (e.g. regression coefficient) AND variation (e.g. standard deviation) or associated estimates of uncertainty (e.g. confidence intervals)
☒	☐	For null hypothesis testing, the test statistic (e.g. *F*, *t*, *r*) with confidence intervals, effect sizes, degrees of freedom and *P* value noted<br>*Give P values as exact values whenever suitable.*
☒	☐	For Bayesian analysis, information on the choice of priors and Markov chain Monte Carlo settings
☒	☐	For hierarchical and complex designs, identification of the appropriate level for tests and full reporting of outcomes
☒	☐	Estimates of effect sizes (e.g. Cohen's *d*, Pearson's *r*), indicating how they were calculated

Our web collection on statistics for biologists contains articles on many of the points above.

Software and code

Policy information about availability of computer code

Data collection	No software was used to collect data
Data analysis	MiSeq reporter v2.6 - MiSeq Reporter is a pre-installed software tool that performs secondary data analysis after a MiSeq System sequencing run.<br><br>Trimmomatic v0.36 - A fast multithreaded tool that performs a variety of useful trimming tasks for illumina paired-end and single ended data.<br><br>bwa 0.75 - a software package for mapping low-divergent sequences against a large reference genome<br><br>Samtools v0.1.19 - a suite of programs for interacting with high-throughput sequencing data.<br><br>ivar v1.3.1 - is a computational package that contains functions broadly useful for viral amplicon-based sequencing.<br><br>Nextclade Web v1.10.1 - a tool that identifies differences between your sequences and a reference sequence used by Nextstrain. It performs viral genome alignment, mutation calling, clade assignment, quality checks and phylogenetic placement<br><br>PANGOLIN v3.1.17 and PANGOLEARN v2021-12-06 were used to classify consensus genomes into pango lineages<br><br>Nextstrain pipeline v3.0.6 (Hadfield, 2018)- an open-source project to harness the scientific and public health potential of pathogen genome data.

figtree v1.4.4 (Rambaut, 2018) - a graphical viewer of phylogenetic trees and as a program for producing publication-ready figures

For manuscripts utilizing custom algorithms or software that are central to the research but not yet described in published literature, software must be made available to editors and reviewers. We strongly encourage code deposition in a community repository (e.g. GitHub). See the Nature Portfolio guidelines for submitting code & software for further information.

Data

Policy information about availability of data

All manuscripts must include a data availability statement. This statement should provide the following information, where applicable:

- Accession codes, unique identifiers, or web links for publicly available datasets
- A description of any restrictions on data availability
- For clinical datasets or third party data, please ensure that the statement adheres to our policy

Assembled SARS-CoV-2 genomes from this study were uploaded to www.gisaid.org as FASTA files and their GISAID epi isl numbers are shown in Supplementary Data 1. Other source raw data for this study are provided as the supplementary dataset: https://doi.org/10.6084/m9.figshare.20085506.v1

Human research participants

Policy information about studies involving human research participants and Sex and Gender in Research.

Reporting on sex and gender	There was no gender categorization. Samples were tested for SARS-CoV-2 as received from the sampling sites.
Population characteristics	There were no prior covariants. All samples from cases suspected to have COVID-19 were analyzed.
Recruitment	This study was performed as part of public health surveillance approved by the Kenya government through the Ministry of Health as part of response to the COVID-19 pandemic.
Ethics oversight	The Scientific and Ethical Research Unit (SERU) of Kenya Medical Research Institute (KEMRI) approved a protocol to allow whole genome sequencing (SERU 4035).

Note that full information on the approval of the study protocol must also be provided in the manuscript.

Field-specific reporting

Please select the one below that is the best fit for your research. If you are not sure, read the appropriate sections before making your selection.

☒ Life sciences ☐ Behavioural & social sciences ☐ Ecological, evolutionary & environmental sciences

For a reference copy of the document with all sections, see nature.com/documents/nr-reporting-summary-flat.pdf

Life sciences study design

All studies must disclose on these points even when the disclosure is negative.

Sample size	Sample size was not necessary. The study analyzed all SARS-CoV-2 genomes generated at our Laboratory (from March 2020 to January 2022).
Data exclusions	SARS-CoV-2 samples testing positive were selected for whole genome sequencing if they had cycle threshold (Ct) <33. Of the 1089 COVID-19 nasal specimens that passed the threshold, 45 were dropped because they did not pass the threshold required for assigning Pango lineages. Ten additional samples were dropped because they lacked date of collection. The remaining 1034 genomes were used to monitor the evolution of SARS-CoV-2 lineages across the five COVID-19 waves. Of the 1034 genomes that were used for lineage assignments, only 969 with genome lengths >27000 bp could be used for phylogenetic analysis.
Replication	Replication is in built in whole genome sequencing. In our case, each base had to have a minimum of 10 reads to be included in the analysis.
Randomization	Randomization was not appropriate. There were no experimental groups.
Blinding	Blinding was not necessary for this study. There were no experimental group to warrant concealment.

Reporting for specific materials, systems and methods

We require information from authors about some types of materials, experimental systems and methods used in many studies. Here, indicate whether each material, system or method listed is relevant to your study. If you are not sure if a list item applies to your research, read the appropriate section before selecting a response.

Materials & experimental systems

n/a	Involved in the study
☒	☐ Antibodies
☒	☐ Eukaryotic cell lines
☒	☐ Palaeontology and archaeology
☒	☐ Animals and other organisms
☒	☐ Clinical data
☒	☐ Dual use research of concern

Methods

n/a	Involved in the study
☒	☐ ChIP-seq
☒	☐ Flow cytometry
☒	☐ MRI-based neuroimaging
